# Partridge and embryonated partridge egg as new preclinical models for candidiasis

**DOI:** 10.1038/s41598-021-81592-y

**Published:** 2021-01-22

**Authors:** Hadi Tavakkoli, Ahmad Khosravi, Iraj Sharifi, Zohreh Salari, Ehsan Salarkia, Reza Kheirandish, Kazem Dehghantalebi, Maziar Jajarmi, Seyedeh Saedeh Mosallanejad, Shahriar Dabiri, Alireza Keyhani

**Affiliations:** 1 Department of Clinical Science, School of Veterinary Medicine, Shahid Bahonar University of Kerman, 22 Bahman Boulevard, Pajouhesh Square, Kerman, 7616914111 Iran; 2 Leishmaniasis Research Center, Kerman University of Medical Sciences, 22 Bahman Boulevard, Pajouhesh Square, Kerman, 7616914115 Iran; 3 Obstetrics and Gynecology Center, Afzalipour School of Medicine, Kerman University of Medical Sciences, Kerman, Iran; 4 Department of Pathobiology, Faculty of Veterinary Medicine, Shahid Bahonar University of Kerman, Kerman, Iran; 5 Afzalipour School of Medicine and Biochemistry Department, Kerman University of Medical Sciences, Kerman, Iran; 6 Afzalipour School of Medicine and Pathology and Stem Cells Research Center, Kerman University of Medical Sciences, Kerman, Iran

**Keywords:** Fungi, Fungal pathogenesis

## Abstract

*Candida albicans* (*C. albicans*) is the most common cause of candidiasis in humans and animals. This study was established to a new experimental infection model for systemic candidiasis using partridge and embryonated partridge egg. First, we tested the induction of systemic candidiasis in partridge and embryonated partridge egg. Finally, interaction between virulence factors of *C. albicans* and Bcl-2 family members was predicted. We observed that embryonic infection causes a decrease in survival time and at later embryonic days (11–12th), embryos showed lesions. Morphometric analysis of the extra-embryonic membrane (EEM) vasculature showed that vascular apoptotic effect of *C. albicans* was revealed by a significant reduction in capillary area. In immunohistochemistry assay, low expression of Bcl-2 and increased expression of Bax confirmed apoptosis. The gene expression of Bax and Bcl-2 was also altered in fungi-exposed EEM. Our*in silico* simulation has shown an accurate interaction between aspartic proteinase, polyamine oxidase, Bcl-2 and BAX. We observed that the disease was associated with adverse consequences, which were similar to human candidiasis. Acquired results support the idea that partridge and embryonated partridge egg can be utilized as appropriate preclinical models to investigate the pathological effects of candidiasis.

## Introduction

Studies on pathogenic infections have been conducted using rat, mice and rabbit as experimental models since pathogenic agents were injected and successfully reproduced clinical symptoms in those models. The mentioned models were also accompanied by in vitro assessments when the symptoms were complicated or the experiment could not be made in animal models due to ethical reasons. The animal models have also been used principally to test treatments with drugs, pharmacokinetics and immunotherapy ^1–3^.

The alternative models based on non-mammalian animals were described using fruit flies (Drosophila melanogaster), the larvae of the moth *Galleria mellonella* and the free-living nematode *CaenorhabditisElegans*^3–6^. Recently, the chicken egg and embryo have also been considered as the laboratory models for experimental investigations^7–9^. To reduce the use of mammalian or non-mammalian animals in medical experimentation, alternative in vivo and in vitro models are increasingly necessary options.

*Candida albicans* (*C. albicans*) is the most common cause of fungal infections in humans ^10,11^. It generally colonizes in regions such as the genitourinary tract, gastrointestinal track, lower respiratory and oropharynx. Candidemia is the fourth most common cause of nosocomial bloodstream infections in the USA and in Europe and has a significant impact on hospitalization cost and patient outcome ^12,13^. An increasing incidence of fungal infections with *Candida spp*. has been noted in immunocompromised patients such as those staying in ICUs, postsurgical and neutropenic patients ^14,15^. The systemic candidiasis has also occurred in patients with malignant diseases in recent years ^16–18^. Different reports in the literature have documented that systemic candidiasis in premature infants is frequently fatal or associated with significant morbidity in survivors ^19–21^. In this respect, introduction of newer in vivo preclinical model for precise studying of the candidiasis, its pathogenesis and treatment is an important and valuable issue nowadays.

In recent years, the partridge industry has experienced remarkable development and chukar partridge has been widely raised industrially in many parts of the world^22,23^. As a research subject, chukar partridge has certain advantages as follows:

Modest size of the bird simplifies housing in laboratory animal facilities and reduced feed costs. The bird handling is easy and fulfilling its nutritional requirement is not a difficult task or expensive. The chukar partridge is not very sensitive to environmental conditions and it can be easily reared in floor and cage system. Furthermore, the modest size of breeding adults facilitates breeding in the laboratory. The female partridges generally lay 3–5 eggs per week and under favorable environments, they produce for long periods. Their eggs can be grown in large batches in incubators, and allow the effects of particular substances on embryonic development to be studied. The partridge embryo is also an amniote and it presents significant experimental advantages for the study of amniotes similar to those of humans. Unlike mammillaria, partridge embryo can be easily studied and manipulated as they monitor by removing a small section of the eggshell. This has made it possible to follow their developmental stages via time-lapsed video-microscopy^24–28^. Therefore, we chose the chukar partridge and embryonated partridge egg so that they could be used as laboratory models in areas that are easy to access.

On the other hand, in the past decade, some species of Phasianidae family (e.g. Japanese Quail), have become important experimental animals for scientific researches. They are used extensively in physiology, nutrition, genetics, embryology, toxicology, pathology and endocrinology researches^29,30^. Chukar partridge has also classified into the Phasianidae family. Considering this property, a major advantage that seems to be very promising is the fact that it is possible to achieve a new experimental model for scientific investigations.

In this context, the objective of this study was to establish new experimental infection models for systemic candidiasis and its pathogenesis using partridge and embryonated partridge egg. The present study was aimed to answer the following questions:

I. Does *C. albicans* by human origin, cause systemic infection in the partridge and partridge embryo?
II. What are the clinical signs, gross and histopathological lesions of systemic candidiasis in partridge model?
III. Does *C. albicans* cause apoptotic effect in vessels?
IV. Does *C. albicans* alter the expression of proteins and genes which have important roles in vascular apoptosis?
V. How is the affinity between *C. albicans* virulence factors and apoptotic-regulator proteins?

## Results

The results are explained in terms of A) clinical and histopathological signs and lesions following systemic candidiasis in partridge model, B) vascular analysis, IHC and qPCR results from the EEM vasculature and C) in silico results.

(A) Clinical and histopathological signs and lesions following systemic candidiasis in partridge model

### Clinical signs

**Figure 1:**
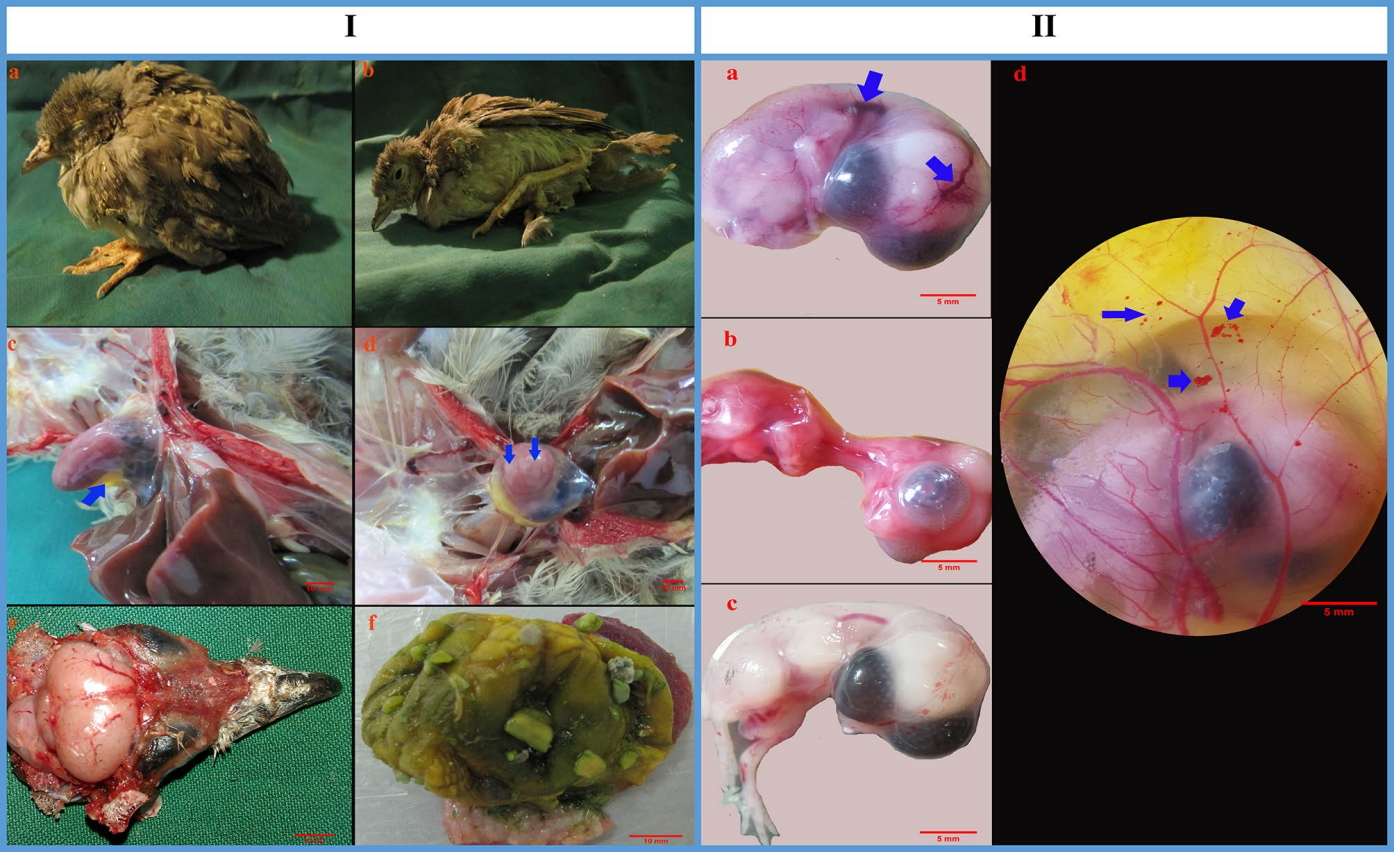
**I** (The *Alectoris chukar* chicks inoculated intravenously with *Candida albicans*. (**a**) The bird is characterized by depression, lethargy and ruffled feathers. (**b**) Following the progression of the disease, infected bird is unable to stand. (**c**) Hydro pericardium is seen in heart (blue arrow). (**d**) White necrotic foci are seen in myocardium (blue arrows). (**e**) Congestion is seen on the brain surface. (**f**) The gizzard is devoid of food in debilitated bird). **II** The partridge embryo model and its extra-embryonic membrane *C. albicans* inoculation. (**a**) Vascular congestion (blue arrows) or (**b**) general congestion is located on the embryo skin. (**c**) Normal embryo did not show any gross lesion. (**d**) Vascular disruption is occurred on the partridge’s EEM model witch demonstrated by haemorrhages (blue arrows).

The infected partridges began to exhibit the clinical signs on the second day of infection. The early symptoms manifested with multiple adverse health outcomes which included depression, lethargy and ruffled feathers (Fig. 1I-a). Following the progression of the disease, some infected birds were unable to stand and maintained their posture with drooping of the wings (Fig. 1I-b). Neurological disturbance such as spasmodic tremors in the head was also seen in four sick birds, mainly on the fourth day after infection. No mortality occurred during the experimental period in the infected group; however, two birds were culled due to severe debilitation on days 3 and 4 following infection. No clinical signs were observed in any of the control birds.

### Cloacal temperature

The normal cloacal temperature of partridge chicks, before the inoculation of *C. albicans*, was 41.3 ± 0.3 °C. An increase in the cloacal temperature was a primary response to infection. The temperature began to increase from normal value 30 h after infection. It continued to increase for another 24 h (≥ 42 °C) and remained almost constant for 3–4 days post-infection. The mentioned temperature (≥ 42 °C) was not observed in any of the control birds. It was also significant after statistical analysis (control bird = 41.29 ± 0.17, infected birds = 41.95 ± 0.07, *p* = 0.002).

### Body weight

**Figure 2:**
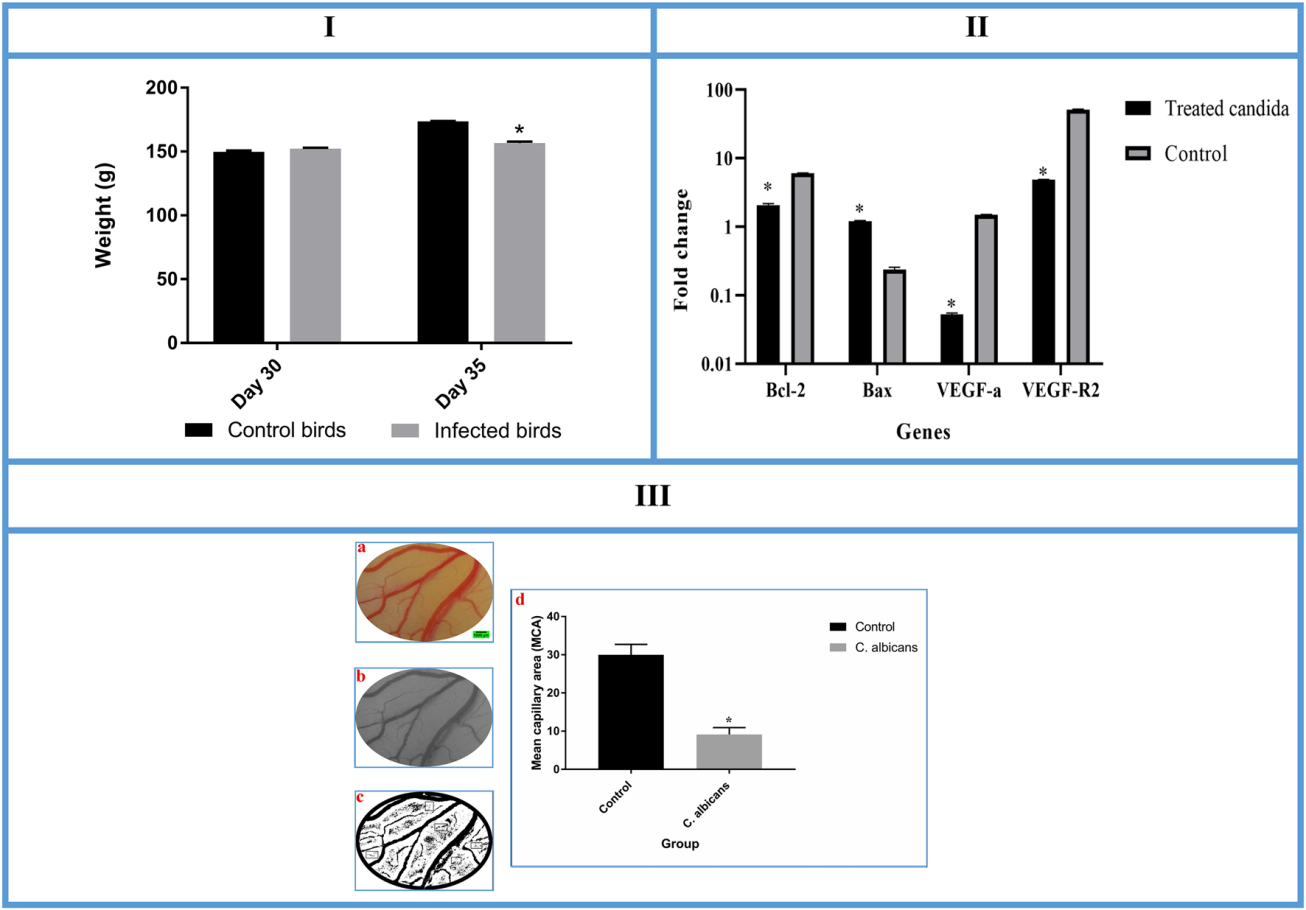
**I** (The body weight of the *Alectoris chukar* chicks at the start of experiment (day 30) and 5 days after infection (day 35) with comparison between control and *Candida albicans* infected birds. The infected birds showed significant weight loss compared to controls at the end of the experiment (error bars show standard error of mean; *p < 0.05).) **II**:(Expression levels of apoptotic-regulator genes following *Candida albicans* treatment. The expression level of Bax mRNA in the partridge extra-embryonic membranes is increased but the expression levels of Bcl-2, VEGF-A and VEGF-R2 are decreased in the LM-treated groups compared to controls. (Error bars show standard error of mean).**III** (The mean capillary area (MCA) analysis on the partridge’s extra-embryonic membrane. (**a**) A constant area (125 mm2) is extracted from captured image. (**b**) Extracted area has been converted to a binarized image. (**c**) Five defined areas (rectangular) without any branch vessels are selected and the percentage of the areas containing black pixels is quantified for the MCA. The black pixels of the image indicate the red color, or blood, in the original image. (**d**) MCA is significantly decreased in the fungus inoculated group compared to the control (error bars show standard error of mean; *p < 0.000, *T* test).

Figure 2I shows the body weight of infected and control partridges at the beginning of the experiment (day 30) and 5 days after infection (day 35). The body weights of the infected partridges were significantly different from those of the control birds at the end of the experiment. A marked decrease was observed in the growth rate of the inoculated birds after injection of the *C. albicans* (152.2 ± 0.75 g at day 30; 156.5 ± 1.3 g at day 35, *p* = 0.033). The control partridges had normal weights (149.8 ± 1.03 g at day 30; 173.6 ± 0.34 g at day 35).

### Gross findings

Gross lesions associated with systemic candidiasis were observed in the kidneys, liver, lungs, heart and brain. Congestion was noticed in some internal organs including the kidneys, liver and lungs of the affected birds. The kidneys and liver were swollen. In some affected birds, hydropericardium with white necrotic foci occurred in myocardium (Figs. 1I-c,d). Congestion of the brain surfaces was evident in birds showing neurological disturbance (Fig. 1I-e). The alimentary tract of most cases was devoid of food (Fig. 1I-f). Muscular atrophy was observed in debilitated birds; however, the skeletal structure appeared normal.

### Histopathological findings

**Figure 3:**
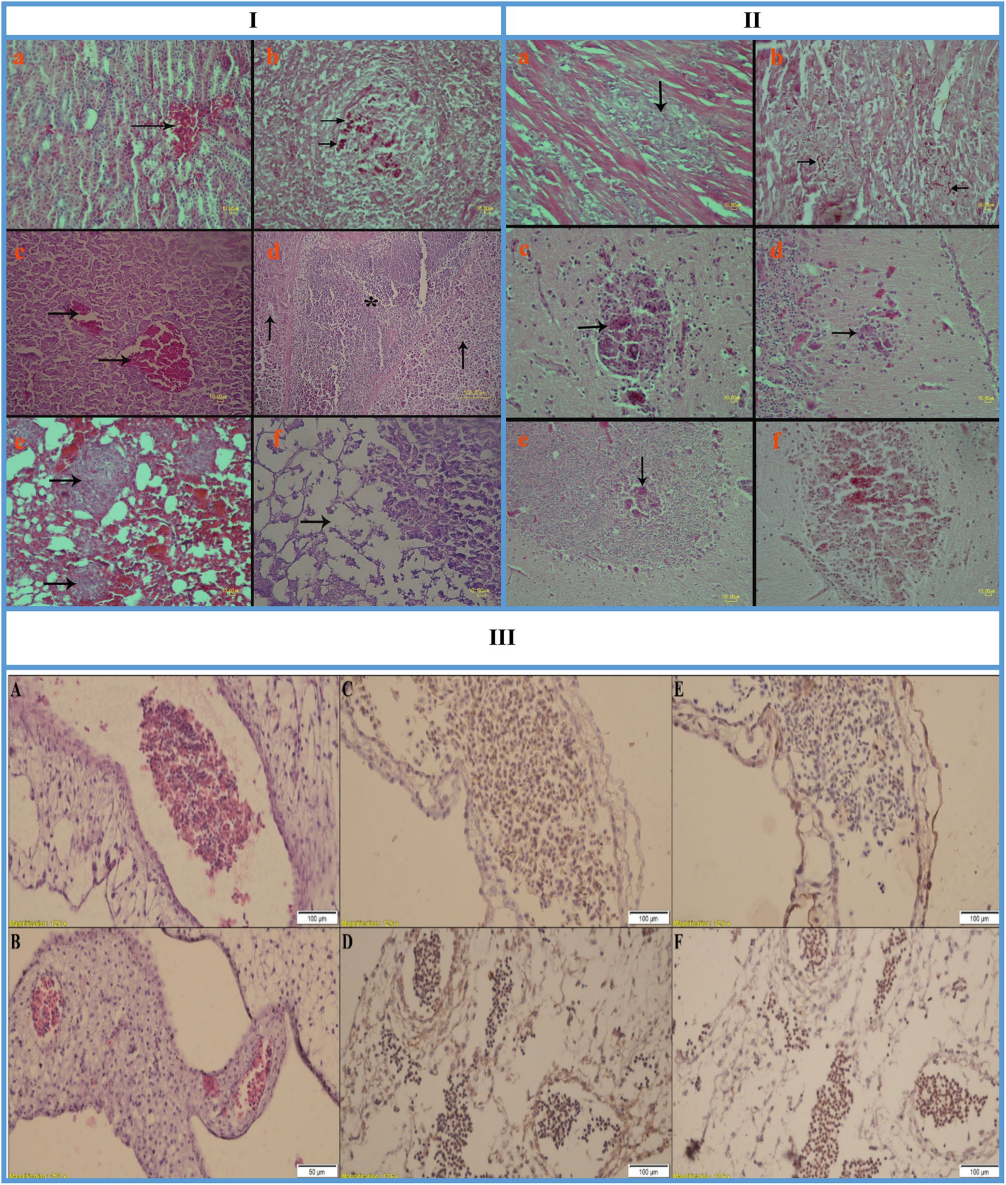
**I** (Histopathological lesions encountered in *Alectoris chukar* inoculated with *Candida albicans.* (**a**) Congestion (arrow) is seen in kidney, H&E. (**b**) Mycelial and yeast forms of *Candida albicans* (arrows) are seen in spleen, PAS. (**c**) Congestion (arrows) is seen in liver, H&E. (**d**) Central vein dilatation (asterix) and hepatocellular necrosis (arrows) are seen in liver, H&E. (**e**) Congestion and granulomas (arrows) are seen in lung, H&E. (**f**) Degeneration and necrosis (arrow) are noticed in the exocrine portion of the pancreas, H&E). **II** (Histopathological lesions encountered in *Alectoris chukar* inoculated with *Candida albicans.* (**a**) Myocardial necrosis with mononuclear cell infiltration (arrow) are seen in myocardium, H&E. (**b**) Mycelial and yeast forms of *Candida albicans* (arrows) are seen in myocardium, PAS. (**c**) Granuloma (arrow) is seen in cerebrum, H&E. (**d** and **e**) Granuloma (arrow) is seen in the molecular and granular layer of cerebellum, respectively, H&E. (**f**) Granuloma with mycelial and yeast forms of *Candida albicans* are seen in cerebrum, PAS.) **III**:(Effects of systemic candidiasis in partridge on vascular apoptosis and blood vessel system (× 40). (**A**) H&E stained vessels in the control embryo: the embryo with normal blood vessel and adequate distribution of leukocytes. RBC's endothelial living cells seem normal. (**B**) H&E stained vessels in the infected embryo: decreasing and compassing of embryonic vasculature compared with the control. (**C**) IHC staining with Bax in the control embryo: scattered Bax positive blood cells and rarely positive endothelial and stromal cells. (**D**) IHC staining with Bax in the infected embryo: compassed vessels with more positive stained leukocytes and stromal cells, compared with the control group. (**E**) IHC staining with Bcl-2 in the control embryo: compassed with Bcl-2 IHC staining more cells are stained. (**F**) IHC staining with Bcl-2 in the infected embryo: compassed vessels with less positive stained leukocytes and stromal cells, compared with the control group).

Histological lesions were observed in the kidneys, liver, spleen, lungs, pancreas, heart and brain of the infected birds. Varying degrees of congestion were detected in some internal organs including the kidneys, liver and lungs (Fig. 3I-a, c and e). In some cases, granulomas were evident in the lungs (Fig. 3I-e), spleen (Fig. 3I-b) and brain (Fig. 3II-c to f). Histologically, granulomas were composed of both mycelial and yeast forms of *C. albicans* with areas of necrosis and inflammatory cell infiltration (particularly the macrophages). Lesions of the liver included congestion, hepatocellular degeneration, necrosis, central vein dilatation, bile duct hyperplasia and vascular thrombosis (Fig. 3I-c and d). The pancreas showed degeneration and necrosis in the exocrine portion (Fig. 3I-f). Myocardial fiber necrosis with infiltration of mononuclear cells, particularly the macrophages, was noticed in myocardium (Fig. 3II-a and b). In the brain, gliosis, edema, neuronal ischemic cell change and granuloma formation in the cerebrum, molecular/granular layer of the cerebellum and midbrain were observed (Fig. 3II-c to f).

### Susceptibility of the partridge embryo model to systemic candidiasis

To confirm the susceptibility of the partridge embryo model to *C. albicans* by human origin, we chose the 10th embryonic day. We observed that embryonic infection causes a decrease in survival time, and at later embryonic days (the 11–12th) of infection, embryos showed gross pathological lesions. The lesions were generally noticed as vascular congestion (Fig. 3II-a) or general congestion (Fig. 3II-b), which was located on the skin. Vascular disruption also occurred on the partridge EEM model that was expressed by haemorrhages (Fig. 3II-d).

(B) Vasculature analysis, IHC and qPCR results from EEM vasculature

As described previously, the EEM partridge modelwas used to evaluate the effect of *C. albicans* on vascular apoptosis. The results were as follows:

### Mean capillary area

The response to *C. albicans* inoculation onto the partridge EEM is presented in Fig. 2III-d. There was a significant decrease in MCA of the vasculature of the treated group (control group, 29.98 ± 2.72; infected group, 9.12 ± 1.80; *p* = 0.034).

### Immunohistochemistry results

In order to prove vascular apoptosis in the partridges’ EEM vasculature, H&E and IHC staining were performed to detect apoptotic cells and their components. As illustrated in Fig. 3III, in *C. albicans* infected group, Bcl-2 on leukocytes, endothelial and stromal cells wereless intensely stained compared with Bax.

### Gene expression results

The expression levels of apoptotic-regulator genes in *C. albicans*infected EEM were determined by qPCR. Our results showed increased expression of Bax genes and reduced expression of Bcl-2, VEGF-A and VEGF-R2 (Fig. 2II).

(C) *In silico* results

In silico assay, as mentioned earlier, was applied to investigate the interaction between *C. albicans* virulence factors (ASP, PAO and TRY) and apoptotic-regulator proteins (Bcl-2 and Bax). The results are presented below.

### Structural parameters of simulated proteins

**Figure 4:**
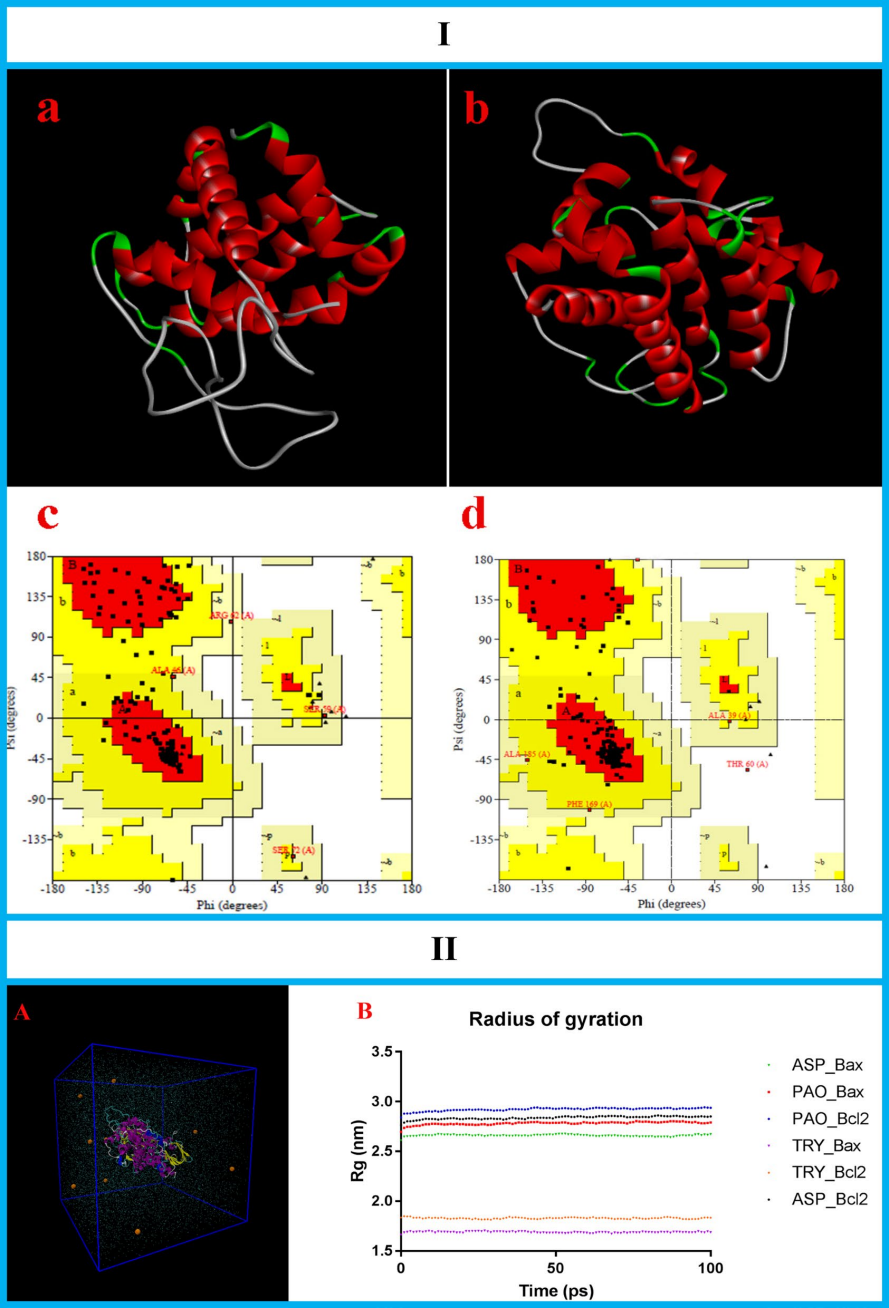
**I (**Structural parameters of simulated proteins (**a** and **b**), Protein models of Bcl-2 and Bax (*Gallus gallus*), respectively The structures of Bcl-2 and Bax (*Gallus gallus*) were constructed using SWISS-MODEL (https://swissmodel.expasy.org/). (**c** and **d**) Ramachandran plots for protein model of Bcl-2 and Bax (*Gallus gallus*), respectively. Ramachandran plot was created using PROCHECK server,https://servicesn.mbi.ucla.edu/PROCHECK/). **II** (Molecular dynamics simulations (**A**) Simulated proteins solvated in SPC water (cyan) and sodium ions (orange). (**B**) Radius of gyration values for simulated proteins. By calculating the radius of gyration, it is revealed that the proteins remain stable, over the course of runs. *ASP* aspartic proteinase, *PAO* polyamine oxidase, *TRY* tryptophol). Molecular dynamics simulation was performed using the GROMACS 5.4.1 package http://www.gromacs.org/.

The bioinformatics tools were applied to construct the structures of the apoptotic-regulator proteins (Bcl-2 and Bax, *Gallus gallus*) (Fig. 4I-a and b). The qualities of constructed models were checked by Ramachandran plots (Fig. 4I-c and d). Ramachandran results for Bcl-2 and Bax were respectively as follows: 88.1% and 87.2% of the residues located in most favored regions, 9.4% and 9.9% of the residues located in additional allowed regions and 2.5% and 2.3% of the residues located in generously allowed regions. The Ramachandran plot is a fundamental tool in the analysis of protein structure. It locates the amino acid residues of the simulated models Ramachandran^31^. A good quality Ramachandran plot has over 85–90% in the most favored regions. Ramachandran plots of simulated proteins (Bcl-2 and BAX) have 88.1% and 87.2% of residues in the most favored regions; therefore, these data exhibited that good quality models were simulated.

The structural parameters of the simulated Bcl-2 were as follows: molecular weight = 21,422.02, total number of atoms = 2954, total number of amino acids = 194, number of negatively charged residues (Asp + Glu) = 22 and the number of positively charged residues (Arg + Lys) = 17.

The conformational parameters of the simulated Bax were as follows: molecular weight = 21,666.16, total number of atoms = 3085, total number of amino acids = 196, number of negatively charged residues (Asp + Glu) = 19 and number of positively charged residues (Arg + Lys) = 17.

### Molecular docking

After constructing the 3D structures of Bcl-2 and Bax, active binding pockets were determined using CASTp program. The volumes of the pockets were 6391.543 and 7094.497 for Bcl-2 and Bax, respectively.

**Figure5:**
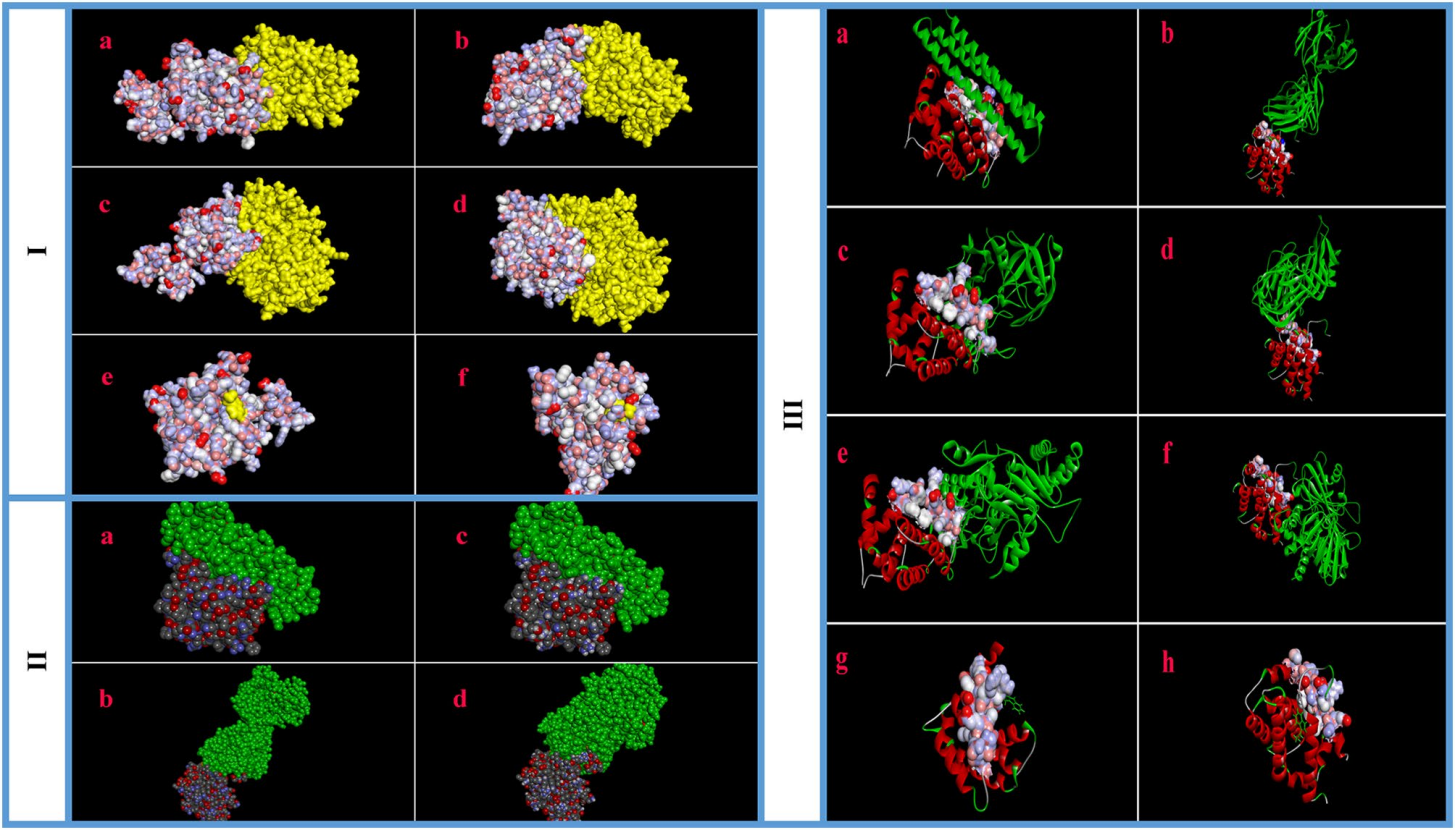
**I** (The docking results between apoptotic-regulator proteins (yellow/right) and virulence factors of *C. albicans* (left). The van der Waals surface of each atom in the protein structure is demonstrated. (**a**) Successful docking between ASP and Bcl-2. (**b**) Protein–protein interaction \ successfully occurred between ASP and Bax. (**c**) Successful docking between PAO and Bcl-2. (**d**) Protein–protein interaction occurred between PAO and Bax. (**e**) The docking between TRY and Bcl-2 with low affinity. (**f**) Protein–protein interaction occurred between TRY and Bax with low affinity. Bax; simulated Bax of *Gallus gallus*. Bcl-2; simulated Bcl-2 of *Gallus gallus*. *ASP* aspartic proteinases: virulence factor of *C. albicans. PAO* polyamine oxidase: virulence factor of *C. albicans. TRY* tryptophol: virulence factor of *C. albicans.* **II** (Validation stage through self-dockings between apoptotic-regulator proteins (left) and their original ligands (green/right) (**a**) 5JSN; Crystallographic structure of human Bcl-2 in complex with specific inhibitor. (**b**) 5W5X; crystallographic structure of human Bax in complex with specific activator. (**c** and **d**) Binding orientations predicted by the self-dockings are in agreement with the binding orientations confirmed by experiment.) **III** (Cross docking between *C. albicans* virulence factors (left) and apoptotic-regulator proteins (green/right). Similar residues located in the binding sites of 5JSN and 5W5X facing toward their ligands are illustrated (white/blue) (**a**) 5JSN; Crystallographic structure of human Bcl-2 protein in complex with specific inhibitor. (**b**) 5W5X; crystallographic structure of human Bax protein in complex with activator. (**c**) Successful cross-docking between ASP and 5JSN. (**d**) Successful cross-docking between ASP and 5W5X. (**e**) Successful cross-docking between PAO and 5JSN. (**f**) Successful cross-docking between PAO and 5W5X. (**g**) Cross-docking between TRY and 5JSN with low affinity. (**h**) Cross-docking between TRY and 5JSN with low affinity. ASP; aspartic proteinases: virulence factor of *C. albicans. PAO* polyamine oxidase: virulence factor of *C. albicans. TRY* tryptophol: virulence factor of *C. albicans.* Figure 5.I, II and III were created using the HEX 8.0.0 software http://hex.loria.fr/dist/index.php).

The dockings of ASP, PAO and TRY with Bcl-2 and Bax were performed using the HEX program. The PDB file format of receptors and ligands were uploaded in HEX for energy minimization and structure refinement and then, the highest scoring conformations (lowest energy) were selected. The results are described below:

The docking between ASP and Bcl-2 was successful, with a significant score (E-total score: − 564.43 kcal/mol). The 3D docking result is demonstrated in Fig. 5I-a. When searching for binding residues on the surface of ASP, we detected that VAL, GLU, SER, VAL, ASN, ARG, GLU, MET, ALA, GLN, MET, SER, GLY, GLN, LEU, HIS, LEU and THR were important for the interaction with Bcl-2.

The analysis of docking data showed that ASP also interacted with Bax (E-total score: − 610.11 kcal/mol) by the binding residues including LEU, THR, VAL, GLY, GLY, TRP, MET, ASN, SER, ILE, PRO, ALA, LEU, ALA, CYS, PHE, SER, VAL, ASP, GLN, PHE, SER, GLY and SER (Fig. 5I-b).

As with the case of ASP, the docking between PAO and Bcl-2 was successful with a significant score (E-total score: − 507.77 kcal/mol) (Fig. 5I-c). For PAO, various residues including THR, ALA, HIS, GLY, ARG, PHE, VAL, ALA, VAL, VAL, GLU, GLU, LEU, PHE, ARG, ASP, GLY, ASN, TRP, ILE, GLN, ASP and ASN were predicted to interact with Bcl-2.

After docking, the affinity between PAO and Bax was also high (E-total score: − 526.59 kcal/mol) (Fig. 5I-d) and the protein–protein interaction successfully occurred through the binding residues including ASP, LYS, LEU, ASP, GLN, ASP, GLN, ALA, PHE, ASN, ASP, MET, ILE, ASP, GLY, LEU and VAL.

The analysis of data showed that TRY interacted with Bcl-2 and Bax with low affinity (E-total score: − 143.62 and − 160.53 kcal/mol, respectively) (Fig. 5I-e and f).

### Affinity of *C. albicans* virulence factors for Bcl-2 family from different origin

As pointed out earlier, cross-dockings were done with 5JSN and 5W5X, instead of their specific ligands, to evaluate the affinity of ASP, PAO and TRY for Bcl-2 family proteins that originated from a different species (Human). The results are shown in Fig. 5III and Table 1. The analysis of data revealed that after separating the original ligands from 5JSN and 5W5X, the ASP, PAO and TRY still tended to interact with them. The 5JSN and 5W5X are crystallographic structures of human Bcl-2 and Bax, which interacted with specific inhibitor or activator, respectively. When searching for binding residues on the surfaces of 5JSN and 5W5X faced toward their inhibitor or activator, various residues including ASP, PHE, SER, ARG, ARG, TYR, ARG, ARG, ASP, PHE, ALA, GLU, MET, SER, SER, GLN, LEU as well as SER, GLU, SER, LEU, LYS, ARG, ILE, GLY, ASP, GLU, LEU, ASP, SER, ASN, MET were predicted, respectively (Fig. 5III-a and b). When comparing these results, we found that several of these residues are known to interact with ASP, PAO and TRY and are located near the binding sites of 5JSN and 5W5X with ASP, PAO and TRY, suggesting that ASP, PAO and TRY may alter the activity of 5JSN and 5W5X similar to that of their specific inhibitor or activator. Similar residues, which are located at the binding sites of 5JSN and 5W5X faced toward their ligands, are illustrated in Fig. 5III-c to h (residues in white color).

**Table 1:** Cross-dockings and self-dockings results.

Docking	Bcl-2 family	Virulence factors	E-total (kcal/mol)	Binding affinity
Cross-docking	Bcl-2	ASP	− 612.57	+
Cross-docking	Bcl-2	PAO	− 528.99	+
Cross-docking	Bcl-2	TRY	− 156.31	±
Cross-docking	Bax	ASP	− 502.44	+
Cross-docking	Bax	PAO	− 483.25	+
Cross-docking	Bax	TRY	− 151.07	±
Self-docking	5W5X	Self-ligand	− 391.77	+
Self-docking	5JSN	Self-ligand	− 1029.09	+

+ ; high affinity.

± ; low affinity.

### Self-docking

We assessed the accuracy of the results by a self-docking stage (validation stage). Among the various conformations, the best-docked conformation was selected. Self-docking results for 5JSN and 5W5X are listed in Table 1, and the best conformation for each docking is demonstrated in Fig. 5II. As predicted, the accuracy of the results was proved with an appropriate binding energy. As revealed in Fig. 5II-c and d, the specific ligands of the 5JSN and 5W5X were docked with their receptors by correct orientations.

### Molecular dynamics results

Molecular dynamics simulation was performed to assess the conformational stability of simulated proteins (ASP, PAO, TRY, Bcl-2 and Bax) and their interactions in the simulated body environment. The simulation box is illustrated in Fig. 5II-A. By calculating the radius of gyration, it was revealed that the proteins remain stable, in its compact (folded) form over the course of runs (Fig. 5II-B). The Van der Waal's interactions (Lennard–Jones and Coulomb interactions), solvent accessible surface areas, number of hydrogen bonds and density of structures are presented in Table 2.

**Table 2:** Molecular dynamics simulation results.

Bcl-2 family	Virulence factors	LJ (kJ/mol)	Coul (kJ/mol)	SASA (nm^2^)	NHB	Density (kg m^−3^)
Bcl-2	ASP	− 589.92	− 985.23	288.66	26.66	1005.11
Bcl-2	PAO	− 1265.08	− 1722.55	360.71	37.94	1005.93
Bcl-2	TRY	− 57.32	− 42.71	120.68	0.99	1020.46
Bax	ASP	− 505.32	− 839.19	280.85	24.21	1015.24
Bax	PAO	− 1281.96	− 1673.63	357.00	41.90	1013.41
Bax	TRY	− 63.48	− 50.64	114.55	1.07	1025.48

*LJ* Lennard–Jones interaction, *Coul* Coulomb interaction, *SASA* Solvent accessible surface areas, *NHB* Number of hydrogen bonds (average per timeframe).

## Discussion

In this work, we demonstrate that the partridge and embryonated partridge egg are new and effective preclinical infection models for evaluating systemic candidiasis. Moreover, some mechanisms, which could be associated with the pathogenesis of the organism, were assessed. Various disorders and lesions were noticed following systemic candidiasis in partridge.

The clinical signs were seen over a 48–96 h period in the partridge model. It was a variety of adverse health consequences including depression, lethargy, decreased feed and water intake and growth depression. Five days post-infection, the analysis of body weight revealed that *C. albicans* caused severe growth depression. The next indicator of the systemic candidiasis in the partridge model was neurological disturbance. Several types of neural disturbances were observed in the Galliformes: 1) extreme opisthotonus with spasmodic tremors, 2) extreme torticollis with cranial rotation and 3) extreme torticollis which resulted in the head being drawn in a medial-ventral direction ^32^. A spasmodic tremor was seen in the head of some partridge birds on day 4 post-infection. Birds with head tremors exhibited the microscopic appearance of *C. albicans* in different parts of the brain.

Various gross abnormalities and microscopic lesions in multiple tissues were also indicators of the candidiasis in the partridge model. Based on the results of this study, it is deduced that *C. albicans* can affect the kidneys, liver, spleen, lungs, pancreas, heart and brain of the partridge model. We noticed gross and microscopic lesions in these organs, however, further data may be provided on alterations in other organs.

Our results are in agreement with the clinical presentation of bloodstream candidiasis in humans. It generally presents as fever and worsening of clinical status, especially in a patient with risk factors who is not responding to appropriate therapy. When *C. albicans* disseminates in humans, various organs are usually involved with the kidney, liver, spleen, myocardium and eye being the most common, and less frequently the other organs (e.g. lung, joint and bone). Candida infects both meninges and brain parenchymal tissue. The organism induces diffuse encephalopathy with diminished consciousness in which the predominant lesion is micro abscess. The severity of the illness and its profile (acute or chronic) may be conditioned by the size of the inoculums. In the partridge model (our work), 5 × 10^5^cfuof *C. albicans* produces neurocandidiasis on the fourth day after infection.

Complex cellular and molecular processes may be related to the pathogenesis of *C. albicans*. In the current research, details of the vascular apoptotic effect of *C. albicans* were assessed through in vivo (EEM partridge model) and in silico investigations. Some highlights of our findings on the vascular alteration are herein discussed.

Morphometrical analysis of the partridge’s EEM vasculature has revealed that *C. albicans* affects the vascular plexus negatively. The method applied to assess the vascular alteration effect of *C. albicans* was the calculation of the mean capillary area in the captured images. To date, this method has been widely used in vascular analysis ^7,33,34^.

The next aspect to discuss is the considerable alteration in the expression of Bcl-2 and Bax proteins following *C. albicans* treatment. The pathways and mechanisms by which *C. albicans* induced apoptotic activity in vessels have not been identified explicitly. However, regarding the IHC results, which revealed that Bcl-2 was less intensely and Bax was more intensely stained in *C. albicans* treated EEM, we suggest that alteration in the Bcl-2 family members could be one of the mechanisms involved in the vascular apoptotic activity of *C. albicans*. This assumption was also supported by our in silico study in which *C. albicans* virulence factors bound to the active site of Bcl-2 and Bax proteins.

The results of our study verified the low expression of Bcl-2, VEGF-A and VEGF-R2 and increased expression of Bax genes in the fungus-exposed group. It seems that Akt/PKB signaling pathway is involved in this process and consequently the VEGF-A caused a decrease in the expression of Bcl2 and increased the expressions of Bax gene ^35^.

We also exploit a docking assay to reveal more details about the apoptotic activity of *C. albicans*. Docking assay has recently been considered as a valuable technique to elucidate the interaction between receptor and ligand ^36–38^. It is well known that the Bcl-2 family members are critical targets for apoptotic and anti-apoptotic agents ^39,40^. In this regard, an important highlight in our study is the interaction of ASP, PAO and TRY with Bcl-2 and Bax proteins. Our data suggest the modulation of Bcl-2 family members via the binding of *C. albicans* virulence factors, which must be validated by further experiments.

Another highlight of the protein–protein interaction is the quantitative aspect of binding affinity between *C. albicans* virulence factors and Bcl-2 family proteins. Based on docking results, which revealed the lowest scoring energy for ASP (− 564.43 and − 610.11) compared to PAO (− 507.77 and − 526.59) and TRY (− 143.62 and − 160.53), it is predicted that the affinity of ASP for Bcl-2 and Bax is higher than that of PAO and TRY. Therefore, ASP can be regarded as a hopeful target for designing anti-apoptotic agents to alleviate the devastating outcomes of candidiasis.

As explained in the materials and methods section, cross-docking was performed to appraise the possible interaction of ASP, PAO and TRY with Bcl-2 family members of Homo sapiens (5JSN and 5W5X). Concerning their successful interaction, it is worthwhile to note that the virulence factors of *C. albicans* are able to selectively interact with Bax and Bcl-2 proteins originating from species other than the birds (*Gallus gallus*).

To the best of the authors’ knowledge, this is the first study to target different aspects of systemic candidiasis with the help of the partridge model. Our results show that *C. albicans* of human origin not only causes systemic candidiasis in the partridge model, but also exhibits adverse clinical consequences, which were similar to human candidiasis. Therefore, partridge and embryonated partridge egg are powerful and attractive preclinical models to study the pathogenesis of candidia spp. because of cost, ease of handling and technical feasibility. The acquired results also indicate that *C. albicans* alters the normal growth of vessels and changes the expression of apoptotic-regulator proteins and genes. The presented data on vascular alteration activity of *C. albicans* permit us to suggest that this activity is one of the important pathways in the pathogenicity of organism. In this investigation, we also employed the partridge’s EEM model for apoptotic studies. This model offers a promising approach to assess the role of various mechanisms, which are associated with the pathogenesis of the diseases.

## Methods

This section is explained in terms of A) induction of systemic candidiasis in partridge and embryonated partridge egg, B) effect of *C. albicans* on vasculature and C) interactions between *C. albicans* virulence factors and apoptotic-regulator proteins.

### Materials

Partridge chicks (*Alectoris chukar*) as well as fertile partridge eggs, with the average egg-weight of 20.7 ± 0.5 g, were purchased from Hasanzadeh Partridge Farm, Yazd, Iran. In the supplier farm, the partridge breeders were kept under optimal condition of rearing. Paraffin was obtained from Merck, Darmstadt, Germany. Real-time PCR materials were purchased from Qiagen, Chatsworth, CA and Takara Bio, Inc., Shiga, Japan. Various biological databases and bioinformatics tools like National Center for Biotechnology Information (NCBI), RCSB Protein Data Bank, CASTp (http://sts.bioe.uic.edu/castp/), SWISS-MODEL, (https://swissmodel.expasy.org/), PROCHECK (http://servicesn.mbi.ucla.edu/Verify3D/), GROMACS 5.1.4, HEX 8.0.0, ImageJ 1.48 (National Institutes of Health, Bethesda, Maryland, USA), MATLAB (MathworksMatlabR2015a) and Digimizer4.3.0 (MedCalc Software, Mariakerke, Belgium) were used for in silico evaluations.

(A) Induction of systemic candidiasis in partridge and embryonated partridge egg

This section was designed to clarify whether *C. albicans* of human origin can cause systemic disease in the partridge model. In the next step, the clinical signs and pathological lesions of systemic candidiasis were assessed in the partridge model.

### Organism

The PTCC 5027 strain of *C. albicans* was kindly provided by Persian Type Culture Collection Center (PTCC), Iran. It was obtained from a human patient and, based on antigenic properties, is categorized to serotype A. This isolate produces D-arabinolactone oxidase, DNA topoisomerase, aspartic proteinases, aspartyl proteinase, estrogen-binding protein, lanosterol synthase, 2, 3-oxidosqualenelanosterol cyclase, phenethyl alcohol, polyamine oxidase and tryptophol. The inoculum was prepared from the mentioned strain in Sabouraud's dextrose broth (Oxoid, Thermo Fisher Scientific, Basingstoke, UK) at 42 °C for 18 h^32^. The broth culture was then centrifuged (2 min at 2500 g) and the remaining pellet was washed three times with sterile phosphate buffered saline (pH 7.4). Finally, the cell suspension was adjusted to an optical density of 10^6^ cfu/ml ^41^. The cell numbers in inoculum were confirmed by plating serial dilutions on Sabouraud's dextrose agar plates.

### Birds and housing conditions

Day-old chukarpartridge chicks (*Alectoris chukar*) were obtained from a commercial breeder farm (Hasanzadeh Partridge Farm, Yazd, Iran). Upon arrival at the Animal Research Center of Shahid Bahonar University, Kerman, Iran, the birds were housed in an electrically-heated battery cage (Belderchin Damavand Co. PLC-DQSH, Tehran, Iran) at 34 °C for 10 days and then were held in two floor pens. The partridges were kept 30 days for acclimation. The temperature was controlled and gradually reduced from 34 °C on day 1–29 °C on day 30 and remained constant throughout the study. During the first week of brooding, a photoperiod of 23 h/day was maintained and then a photoperiod of 20 h/day was used. The temperature and photoperiod are necessary for raising and managing partridge chick especially during the first 5 weeks of life. Poor or inadequate temperature and photoperiod is stressful for bird and causing a problem in partridge management. Therefore, temperature and photoperiod were supplied for partridge chicks according to recommended procedures^28^.Food and water were provided ad libitum. The food was formulated according to the nutritional requirements of the partridge chicks from which all medications had been omitted. The ingredients of the diet are presented in Table 3^28^. The trial lasted for 35 days.

**Table 3:** Ingredients and chemical composition of diet during the experimental period (0–35 days).

Feed ingredients	%
Corn	49.38
Soybean meal (44% CP)	44.58
Vegetable oil	1.56
Dicalcium phosphate	1.80
Limestone	1.78
NaCl	0.3
DL-methionine	0.10
Vitamin Premix*	0.25
Mineral Premix*	0.25
Total	100
**Analysis**	
Crude protein	24.00
Ca	1.10
P	0.51
Na	0.11
ME, Kcal/kg	2800

*Vitamin/Mineral Premix (Talavang company, Tehran, Iran) supplied per 5 kg: vitamin A, 11,000,000 IU; cholecalciferol, 5,000,000 IU; vitamin E, 7 500 IU; K3, 3000 mg; vitamin B1, 3000 mg; vitamin B2, 8000 mg; niacin, 4000 mg; d-pantothenic acid, 15,555 mg; vitaminB12, 16 mg; folic acid, 2000 mg; biotin, 150 mg, Mn, 120,000 mg; Fe, 40,000 mg; Zn, 100,000 mg; Cu, 16,000 mg; iodine, 125 mg; Se, 300 mg; cholin chloride, 900,000 mg.

### Experimental design

The experiment was performed according to the suggested European Ethical Guidelines for the care of animals in experimental investigations, in line with the guidelines of Kerman University of Medical Sciences and was approved by the Animal Ethics Committee of the Research Council of Shahid Bahonar University, Kerman, Iran (project number D.550.P.835, Ethics number IR.UK.VETMED.REC. 398.014).

At 30 days of age, twenty-four partridge chicks were selected on the basis of their overall appearance and body weight uniformity. Blood samples were randomly collected aseptically from the brachial vein of ten birds and serological tests (Hemagglutination-inhibition tests for H9N2 avian influenza subtype and Newcastle diseases) as well as bacteriological cultures were done to assess the pre-challenge health status of the partridges ^42^. All sampled birds showed negative results in antibody tests. Negative bacteriological cultures were also obtained. The birds were randomly separated into two different groups of 12 birds each. The partridges of the first group were injected intravenously (in brachial vein) with 0.5 ml of prepared suspension of *C. albicans*. The birds of the second group were used as the control and were intravenously inoculated with sterile phosphate buffered saline. Infectious dose was chosen based on our preliminary investigation and experimental study inducing systemic candidiasis in Galliformes^32^. The birds were monitored twice daily during the experimental period (5 days after inoculation). Each bird was evaluated individually and efforts were made so that just one person could be involved in the clinical evaluation of the birds in order to avoid subjectivity in the analysis. Cloacal temperature and body weight were also recorded. Cloacal temperature was measured four times daily using a digital medical thermometer (FT15/1, Beurer Company, Ulm, Germany, with a range between 35.5 and 42 °C, measurement accuracy ± 0.1 °C). Body weight was measured by a digital scale (Sartorius TE212, Germany, with a range up to 200 g, measurement accuracy ± 0. 01 g). On day 35 of the experiment, the birds were necropsied and tissue samples were taken for pathological investigations. After being fixed in 10% neutral buffered formalin, serial sections of paraffin-embedded tissues were prepared and processed routinely for hematoxylin and eosin (H&E) as well as periodic acid-Schiff (PAS) staining. Tissues of the kidneys, liver, spleen, lungs, pancreas, heart and brain were examined.

### Induction of systemic candidiasis in partridge embryo model

Fertile partridge eggs, of the breed *Alectoris chukar*, were incubated in an electrical incubator (General Cocks, Cocksmachine Company, Tehran, Iran) at 37.5 °C and 60% relative humidity. Prior to the fungal inoculation, eggs were checked for embryonic development by candling. On the10th day of incubation, *C. albicans* was inoculated into the chorioallantoic sac and embryos were allowed to develop for a few more days. For *C. albicans* inoculation, embryo viability was confirmed prior to inoculation by candling, and infertile and nonviable embryos were removed. For all embryos, the air cell was marked, and the egg-shell was disinfected with 70% ethanol. A 1–2-mm diameter region of the shell just above the air cell marking was penetrated using an electric drill, and the chorioallantoic sac was inoculated via a 1-ml syringe with a 1-inch, 22-ga needle.

The 10th day of the embryonic stage was chosen based on our preliminary experiment. On the other hand, on the10th day of incubation, the partridge’s chorioallantoic sac is large enough to inoculate an appropriate volume of fungus. The embryos were examined at 3 different time intervals: 24, 48 and 72 h after inoculation. Finally, the embryos were humanely killed by cooling ^43^. The eggs were opened at the blunt end and embryos were removed to study any gross abnormalities on the external body surface The experiment was repeated for a total of two trials to ensure the repeatability of the experiment.

(B) Effect of *C. albicans* on vasculature

In this study, we used the EEM partridge model for evaluating the effect of *C. albicans* on vasculature and apoptotic-related proteins. In recent years, the EEM of *Gallus gallus* has provided a valuable model for in vivo evaluation of the vascular toxicity of the agents ^44,45^. It is also used as an alternative host model for fungal pathogens ^43,46^. Hence, we used the EEM partridge model to investigate the effect of *C. albicans* on the vasculature and apoptotic-related proteins. This effect was assessed via morphometric analysis of vascular pattern from the partridge EEM. Immunohistochemistry (IHC) and quantitative real-time PCR (qPCR) assays were also performed to evaluate the expressions of apoptotic-regulator proteins and genes following fungus treatment. The details are as follows:

### Experimental design and image acquisition

Fertile partridge eggs were incubated at 37.5 °C and 60% relative humidity. On the 10th day, *C. albicans* was inoculated onto the EEM. After infection, the shell was cut with scissors and a window of 15 × 15 mm was opened to allow image acquisition. High-resolution images (5312 × 2988 pixels) were captured from the EEM vasculature. Afterwards, an area of approximately 10 × 10 mm of partridge’s EEM was removed and subjected to IHC staining.

### Vascular pattern analysis

Image analysis was performed using computerized software such as MATLAB (Mathworks Matlab R2015a) and ImageJ 1.48 (National Institutes of Health, Bethesda, Maryland, USA). Initially, a defined area (125 mm2) of the EEM was extracted and the contrast was improved (Fig. 2III-a). Effort has been made to select a constant area in each image. The extracted area was converted to a binarized format (Fig. 2III-b). Then, the areas without any branch vessels were selected for analysis. Five such areas per case were identified and the percentage of the areas containing black pixels were quantified (Fig. 2III-c). The black pixels of the images indicate the red color, or blood, in the original images. The mean of all areas calculated in each image is described as the mean capillary area (MCA) ^33^.

### Immunohistochemistry assay

The samples of the partridge’s EEM were fixed in 10% buffered formalin and embedded in paraffin. Tissue sections were made by the microtome (Slee Germany) and IHC staining was performed for Bcl-2 (mouse monoclonal antibody, American, ID number: PDMO16-lotH147) and Bax (Zytomed Germany, ID number: 502_17990) markers. The expression levels of Bcl-2 and Bax were assessed by counting the stained cells and calculating the mean in 10 high-power fields (400 ×).

### Gene expression

The effect of *C. albicans* on the apoptotic-regulator genes, in vessels, was evaluated by qPCR assessment of relative expression levels of Bax, Bcl-2, VEGF-A and VEGF-R2 genes. Briefly, the total RNA of the partridge EEMs was isolated using the RNeasy mini kit (Qiagen, USA). A nano-drop was used to evaluate the quality of samples (ND-1000, Thermo Scientific Wilmington, DE, USA) and the cDNA was synthesized using a total of 500 ng RNA by RT reagent kit (Takara, Clontech) according to the protocol. Finally, qPCR was performed using a SYBR Green assay (SYBR Premix Ex Ta II; Japan). The specific primers and reference gene sequences are listed in Bellow.

1. Bax (Forward: CACAGGTGCCTACTGTCGTT)
  (Reverse: CACACTGGGATTCTTCCGCT)226 bp.
2. Bcl-2 (Forward: TCGTCGCCTTCTTCGAGTTC)
  (Reverse:CATCCCATCCTCCGTTGTCC)150 bp.
3. VEGF-R2 (Forward: GCCAACTCTATGGCAGAAGC).
  (Reverse: CTGAACACCATGCCACTGTC)86 bp.
4. VEGF-A (Forward: CAATTGAGACCCTGGTGGAC).
  (Reverse: TCTCATCAGAGGCACACAGG)86 bp.
5. HPRT (Forward: GATGAACAAGGTTACGACCTGGA).
  (Reverse: TATAGCCACCCTTGAGTACACAGAG) 103 bp.
6. GAPDH (Forward: CCTCTCTGGCAAAGTCCAAG).
  (Reverse: GGTCACGCTCCTGGAAGATA)176 bp.

Expression levels were calculated in relation to the expression levels of the selected reference gene. GAPDH and HPRT genes were demonstrated to be the most superior and stable genes in the experiments. All tests were performed in duplicate. Using the 2–ΔCt method, the gene expression level was analyzed in fold change.

(C) Interactions between *C. albicans* virulence factors and apoptotic-regulator proteins

In this section, using in silico approaches, we simulated the three-dimensional structure of *Gallus gallus*Bax and Bcl-2 proteins and predicted their interactions with the virulence factors of *C. albicans*. It has been found that some virulence factors of *C. albicans* such as aspartic proteinase (ASP) ^47^, polyamine oxidase (PAO) ^48–50^ and tryptophol (TRY) ^51,52^ can induce apoptosis in hamster, human epithelial cells and particular cell lines. However, the roles of these factors in partridge-vessels apoptosis are not clearly defined. Therefore, we choose these virulence factors for in silico study. The procedures are described as follows:

### Model simulation and assessment

The structures of Bcl-2 and Bax (*Gallus gallus*) were constructed using SWISS-MODEL (https://swissmodel.expasy.org/) based on target-template alignment. The accuracy of the SWISS-MODEL server has been proved previously ^53^. At first, the domain sequence of Bcl-2 (*Gallus gallus*, ID: NP_990670.2) and Bax (*Gallus gallus*, ID: ACR83547.1) were obtained from the NCBI server and known homologous structures were recognized by blasting between the query sequences. In the next step, we retrieved the closest homologous structures of proteins from the Protein Data Bank and identified the partially homologous structures to serve as a template for Bcl-2 and Bax.

The FASTA formats of the target's sequences (*Gallus gallus*) are listed as follows:

 > Bcl-2: NP_990670.2

MAHPGRRGYDNREIVLKYIHYKLSQRGYDWAAGEDRPPVPPAPAPAAAPAAVAAAGASSHHRPEPPGSAAASEVPPAEGLRPAPPGVHLALRQAGDEFSRRYQRDFAQMSGQLHLTPFTAHGRFVAVVEELFRDGVNWGRIVAFFEFGGVMCVESVNREMSPLVDNIATWMTEYLNRHLHNWIQDNGGWDAFVELYGNSMRPLFDFSWISLKTILSLVLVGACITLGAYLGHK.

 > Bax: ACR83547.1

MACEASQDYQIGEALLIGVVRQELMEVMEVTEGNAAPPALPEAKPISNSQDQILVQ LNTIKVIGDKLDQDQAFNDMIDGLVKVADKSSFWKLVEKVFTDGQINWGRIIVLFYS GLSAKMVVARPRIVSDILSLSLDYFKRNLLQWILTVGGWMNSIPALACFSVDQFSGSSMRKYSPYVGVVAFTGGLLLG FIVSRFQKT.

The simulated proteins were further assessed using Ramachandran plot at PROCHECK server https://servicesn.mbi.ucla.edu/PROCHECK/)31.

### Protein–protein interactions

On the protein surfaces, the active binding pockets were predicted based on computational geometry theories using CASTp server (http://sts.bioe.uic.edu/castp/). Following the pocket identification, the interactions of ASP, PAO and TRY with Bcl-2 and Bax proteins were assessed via HEX 8.0.0 software (http://hex.loria.fr/dist/index.php). This software is introduced in the Critical Assessment of Predicted Interactions (CAPRI, http://capri.ebi.ac.uk/) as a high-quality docking program based on the fast Fourier transform approach ^54^.

The cross-dockings were also performed to evaluate the affinity of ASP, PAO and TRY in a way that Bcl-2 and Bax proteins are linked to specific inhibitor or activator. For this purpose, the molecular structures of 5JSN (crystal structure of Bcl-2 in complex with specific inhibitor) and 5W5X (crystal structure of Bax in complex with specific activator) were selected from the Protein Data Bank (Fig. 5III-a and b). After the separation of the ligands from the coordinates of the receptors, the affinity among ASP, PAO and TRY with 5JSN and 5W5X were assessed. Eventually, a validation step was made by self-dockings among 5JSN, 5W5X and their specific ligands. This step was considered as the key of docking accuracy.

### Molecular dynamics simulation

Molecular dynamics simulation was performed using the GROMACS 5.4.1 package http://www.gromacs.org/ to assess the conformational property of simulated proteins (ASP, PAO, TRY, Bcl-2 and Bax) and their interactions in the simulated body environment. Briefly, the docked structures of ASP, PAO and TRY with Bcl-2 and Bax were subjected to the GROMOS 54A7 force field and the water model SPC was used as the solvent. The solvated system was defined in a charge neutralized system by adding ions (Na or Cl ions). The cubic solvent box was considered and solvated by explicit water using the gmx solvate algorithm. The system was minimized for 50,000 steps using the steepest descent algorithm and then simulation was performed at 310 K and 1 bar under periodic boundary conditions. The v-rescale and Parrinello-Rahman algorithms were used for temperature and pressure coupling, respectively ^55,56^. Electrostatic interactions were calculated by the particle-mesh Ewald method ^57^. During the production phase, conformers were stored every 0.002 ps. The stability of the structure during the simulations was investigated by calculating the radius of gyration using the tool gmx gyrate of the GROMACS package. The h-bonds, Van der Waal's interactions, solvent accessible surface area and density of structures were also analyzed.

### Statistical analysis

Statistical analysis was performed using SPSS version 20. The Fisher's exact and repeated measurement tests were used to determine the significant differences in lesion occurrence and the body weight between experimental groups, respectively. The *T* test was applied to assess the significant differences in the MCA values. A *p* value of < 0.05 was considered statistically significant.

## Supplementary Information

Supplementary Information

## Abbreviations

BAX: Bcl2-associated X protein
Bcl: B-cell lymphoma
*C. albicans*: *Candida albicans*
EEM: Extra-embryonic membrane
H&E: Hemotoxylin and eosin
IHC: Immunohistochemistry
PAS: Periodic acid-schiff
PAO: Polyamine oxidase
ASP: Aspartic proteinase
TRY: Tryptophol
