# Supplementary material for: Partridge and embryonated partridge egg as new preclinical models for candidiasis
Source: Sci Rep. 2021 Jan 22;11:2072. doi: 10.1038/s41598-021-81592-y (PMC7822824; doi:10.1038/s41598-021-81592-y)
Supplement: Supplementary file 1 — Supplementary Information [file 41598_2021_81592_MOESM1_ESM.pdf]

S1: Ramachandran plot for protein model of Bax (*Gallus gallus*)

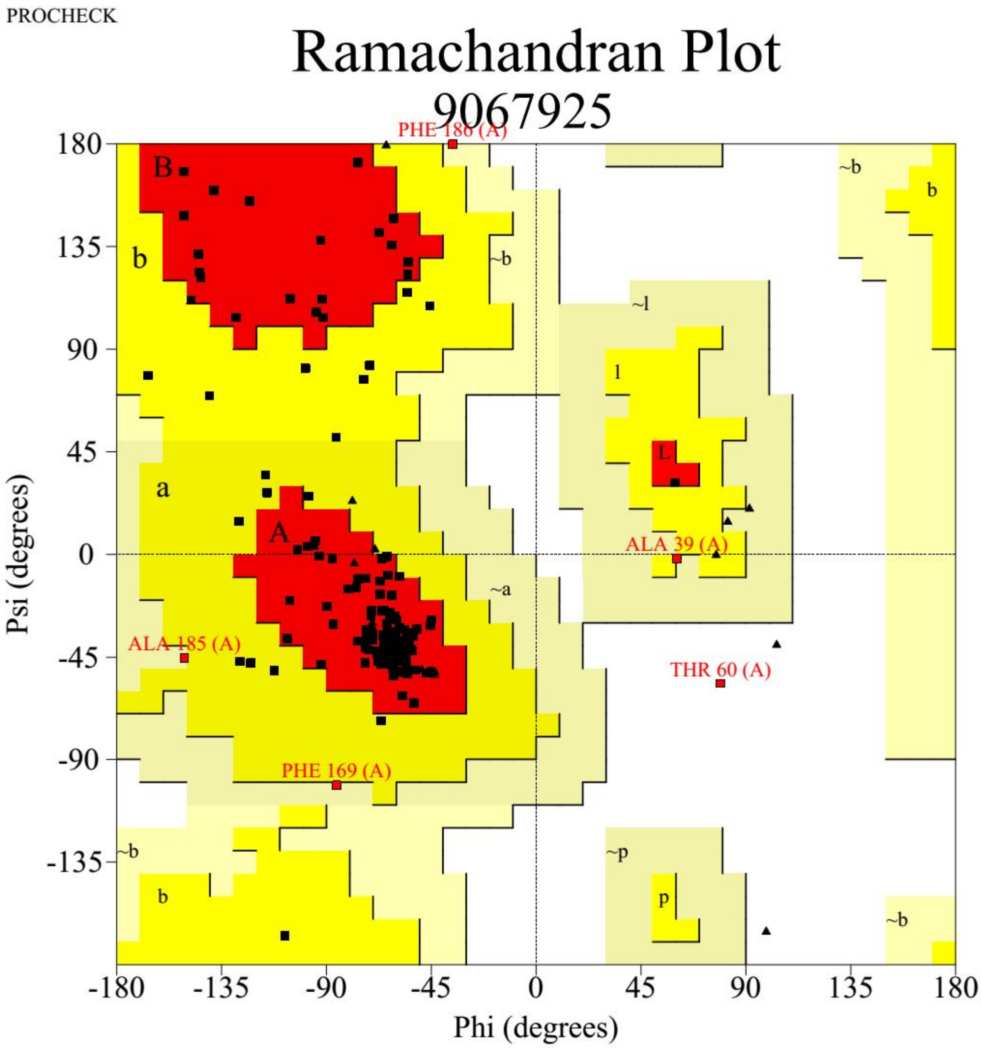

Plot statistics

Residues in most favoured regions [A,B,L]	150	87.2%
Residues in additional allowed regions [a,b,l,p]	17	9.9%
Residues in generously allowed regions [~a,~b,~l,~p]	4	2.3%
Residues in disallowed regions	1	0.6%
	----	------
Number of non-glycine and non-proline residues	172	100.0%
Number of end-residues (excl. Gly and Pro)	2	
Number of glycine residues (shown as triangles)	15	
Number of proline residues	7	

Total number of residues	196	

S2: Ramachandran plot for protein model of Bcl2 (*Gallus gallus*)

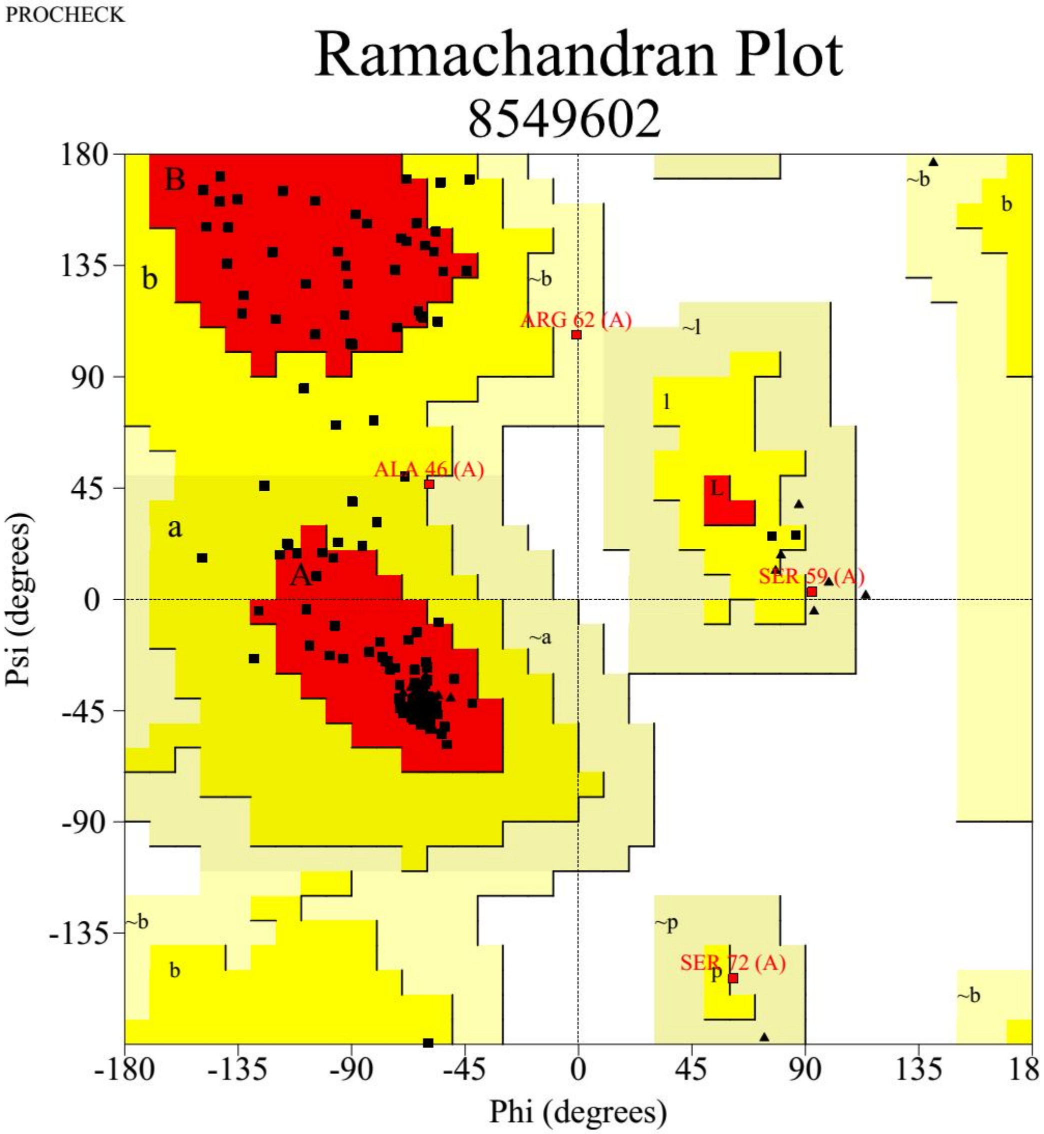

Plot statistics

Residues in most favoured regions [A,B,L]	140	88.1%
Residues in additional allowed regions [a,b,l,p]	15	9.4%
Residues in generously allowed regions [~a,~b,~l,~p]	4	2.5%
Residues in disallowed regions	0	0.0%
	----	------
Number of non-glycine and non-proline residues	159	100.0%
Number of end-residues (excl. Gly and Pro)	1	
Number of glycine residues (shown as triangles)	17	
Number of proline residues	17	

Total number of residues	194	

S3: Radius of gyration values (XVG text file).

```
# This file was created Created by:
#                   :-) GROMACS - gmx gyrate, VERSION 5.4.1 (-:
#
# Executable:   /usr/local/gromacs/bin/gmx
# Data prefix:  /usr/local/gromacs
# Command line:
#   gmx gyrate -s md.tpr -f md.xtc -n index.ndx -o gyrate.xvg
# gmx gyrate is part of G R O M A C S:
#
# GROup of MAchos and Cynical Suckers
#
@    title "Radius of gyration (total and around axes)"
@    xaxis  label "Time (ps)"
@    yaxis  label "Rg (nm)"
@TYPE xy
@ view 0.15, 0.15, 0.75, 0.85
@ legend on
@ legend box on
@ legend loctype view
@ legend 0.78, 0.8
@ legend length 2
@ s0 legend "Rg"
@ s1 legend "Rg\sX\N"
@ s2 legend "Rg\sY\N"
@ s3 legend "Rg\sZ\N"
         0     2.61104     2.08319     2.23548     2.07317
         1     2.64816     2.11073     2.26849      2.1034
         2      2.6507     2.11218     2.27307     2.10339
         3     2.65325      2.1158     2.27546     2.10361
         4     2.65383     2.11553     2.27701     2.10365
         5     2.65421     2.11705     2.27715     2.10293
         6      2.6594     2.11834     2.28321     2.10816
         7     2.66249     2.11552     2.28613     2.11562
         8     2.65857     2.11123     2.28077     2.11586
         9     2.66028     2.10913     2.28498     2.11769
        10     2.65927      2.1113     2.28465     2.11335
        11     2.66615     2.12374     2.29113     2.11119
        12     2.67483     2.12978     2.30033     2.11707
        13     2.67153     2.12435     2.29903     2.11558
        14     2.66741     2.12373     2.29224     2.11319
        15     2.66834     2.12751     2.29039     2.11375
        16     2.66541     2.12873     2.28534     2.11057
        17     2.67136     2.13159     2.28956     2.11815
        18     2.66231      2.1224     2.28159     2.11319
        19     2.66335      2.1224     2.28345      2.1138
        20     2.66437     2.12546     2.28568     2.11087
        21     2.66681     2.12745     2.28692     2.11369
        22     2.66269     2.12256     2.28364     2.11176
        23     2.65814     2.12527     2.27391     2.10808
        24     2.65922     2.13229     2.27407     2.10353
        25     2.66336     2.13917      2.2739     2.10722
        26      2.6592     2.13452     2.27301     2.10235
        27     2.65324     2.12959     2.26557     2.10034
        28     2.65798     2.12947     2.26891     2.10883
        29     2.66179     2.13176     2.27105     2.11381
        30     2.65787     2.12753     2.26534     2.11433
        31     2.65751     2.12577     2.26605     2.11444
        32     2.65684     2.12566     2.26636     2.11252
        33     2.66234     2.13004     2.27272     2.11514
        34     2.65719     2.12782     2.26693     2.11063
        35     2.65638     2.12837      2.2651        2.11
        36     2.65653     2.12944     2.26556      2.1088
        37     2.65998     2.12852     2.27189     2.11161
        38      2.6576     2.12304     2.27203     2.11099
        39     2.65593      2.1221     2.27037     2.10951
        40     2.65973     2.12876     2.27163     2.11102
        41     2.65499      2.1271     2.26552     2.10733
        42      2.6581     2.12919     2.26638     2.11212
        43     2.66342     2.13439      2.2689     2.11756
        44     2.66584      2.1405      2.2699     2.11642
        45     2.66205     2.13449     2.26755     2.11547
        46       2.668     2.13865     2.27439     2.11892
        47     2.66776     2.13566     2.27719      2.1183
```

48	2.66961	2.13529	2.28115	2.11908
49	2.66823	2.13583	2.27837	2.11806
50	2.67307	2.14084	2.28092	2.12247
51	2.67502	2.1389	2.28338	2.12668
52	2.67651	2.14295	2.2829	2.12687
53	2.67778	2.14509	2.28234	2.1285
54	2.67081	2.14349	2.27676	2.11856
55	2.67234	2.14519	2.28003	2.11716
56	2.66542	2.14279	2.27364	2.10902
57	2.66985	2.14645	2.27402	2.11607
58	2.66362	2.14717	2.26636	2.10784
59	2.66269	2.14709	2.2638	2.10832
60	2.66185	2.14338	2.26171	2.11221
61	2.65612	2.13841	2.25744	2.10741
62	2.65765	2.1376	2.25653	2.11303
63	2.652	2.13698	2.25199	2.10428
64	2.65257	2.13672	2.25237	2.10557
65	2.65546	2.13902	2.25509	2.10761
66	2.6574	2.1419	2.25652	2.10806
67	2.65888	2.13839	2.25963	2.11203
68	2.65774	2.13725	2.26134	2.10847
69	2.66143	2.14281	2.26233	2.11108
70	2.65547	2.13995	2.25909	2.10242
71	2.65522	2.14205	2.25746	2.10139
72	2.65085	2.13767	2.25772	2.09454
73	2.65423	2.1429	2.26065	2.0946
74	2.64914	2.14025	2.25357	2.09203
75	2.65004	2.14048	2.25331	2.09438
76	2.65335	2.14282	2.25657	2.09685
77	2.64805	2.13618	2.25104	2.09618
78	2.65156	2.13646	2.25663	2.09874
79	2.64928	2.13562	2.25352	2.09717
80	2.64924	2.13873	2.25341	2.09402
81	2.6511	2.14452	2.25334	2.09288
82	2.64484	2.14128	2.24823	2.08585
83	2.64777	2.14187	2.25144	2.08922
84	2.64643	2.13756	2.25211	2.0895
85	2.64782	2.13987	2.2526	2.09015
86	2.64814	2.14105	2.24667	2.0961
87	2.64151	2.13754	2.24038	2.08968
88	2.64565	2.14281	2.24198	2.09305
89	2.65409	2.14846	2.24757	2.10261
90	2.65692	2.15144	2.25182	2.10214
91	2.66126	2.15484	2.25464	2.10662
92	2.65816	2.15275	2.2524	2.10333
93	2.66088	2.15231	2.25455	2.10834
94	2.65797	2.15254	2.24876	2.10694
95	2.66898	2.15972	2.25214	2.12375
96	2.6729	2.16158	2.25182	2.13205
97	2.66236	2.15035	2.24618	2.12295
98	2.66988	2.15104	2.25789	2.12873
99	2.66577	2.14582	2.254	2.12781
100	2.67175	2.14955	2.25998	2.13271

S4:Van der Waal's interactions (XVG text file).

```
# This file was created by:
#                   :-) GROMACS - gmx energy, VERSION 5.4.1 (-:
#
# Executable:   /usr/local/gromacs/bin/gmx
# Data prefix:  /usr/local/gromacs
# Command line:
#   gmx energy -f md.edr -o vdw.xvg
# gmx energy is part of G R O M A C S:
#
# Gallium Rubidium Oxygen Manganese Argon Carbon Silicon
#
@    title "GROMACS Energies"
@    xaxis  label "Time (ps)"
@    yaxis  label "(kJ/mol)"
@TYPE xy
@ view 0.15, 0.15, 0.75, 0.85
@ legend on
@ legend box on
@ legend loctype view
@ legend 0.78, 0.8
@ legend length 2
@ s0 legend "LJ-SR:protein_chain_A-protein_chain_A2"
    0.000000  -591.805908
    1.000000  -514.316895
    2.000000  -504.176025
    3.000000  -532.226685
    4.000000  -536.961731
    5.000000  -470.354736
    6.000000  -523.232544
    7.000000  -507.464233
    8.000000  -550.922607
    9.000000  -503.581543
   10.000000  -528.745361
   11.000000  -506.396454
   12.000000  -522.193726
   13.000000  -525.778320
   14.000000  -501.990173
   15.000000  -544.985779
   16.000000  -504.361206
   17.000000  -470.616119
   18.000000  -557.565491
   19.000000  -491.093811
   20.000000  -518.102478
   21.000000  -498.132599
   22.000000  -473.704224
   23.000000  -524.326050
   24.000000  -534.721619
   25.000000  -524.417603
   26.000000  -541.945190
   27.000000  -515.849548
   28.000000  -496.035400
   29.000000  -541.817322
   30.000000  -504.755493
   31.000000  -550.524109
   32.000000  -523.860168
   33.000000  -497.934418
   34.000000  -521.151428
   35.000000  -550.165527
   36.000000  -505.435059
   37.000000  -512.259033
   38.000000  -465.708923
   39.000000  -460.675964
   40.000000  -488.304810
   41.000000  -513.472900
   42.000000  -481.526184
   43.000000  -478.643311
   44.000000  -509.828003
   45.000000  -537.974609
   46.000000  -539.162354
   47.000000  -523.228149
   48.000000  -522.823486
   49.000000  -525.453125
   50.000000  -512.562622
```

51.000000	-507.508698
52.000000	-483.935913
53.000000	-468.565063
54.000000	-508.981720
55.000000	-499.709351
56.000000	-484.572174
57.000000	-499.069427
58.000000	-455.674438
59.000000	-535.515076
60.000000	-500.305573
61.000000	-510.933685
62.000000	-480.506287
63.000000	-500.670227
64.000000	-492.095306
65.000000	-524.664551
66.000000	-469.999939
67.000000	-507.436279
68.000000	-478.400848
69.000000	-515.931519
70.000000	-469.857910
71.000000	-527.895203
72.000000	-501.894897
73.000000	-515.315979
74.000000	-495.619873
75.000000	-515.087158
76.000000	-462.721771
77.000000	-487.054962
78.000000	-491.702911
79.000000	-497.475281
80.000000	-510.174927
81.000000	-515.188782
82.000000	-524.826477
83.000000	-500.158478
84.000000	-525.277466
85.000000	-478.398987
86.000000	-511.200958
87.000000	-525.554382
88.000000	-531.833923
89.000000	-511.086273
90.000000	-508.659119
91.000000	-507.786530
92.000000	-522.357056
93.000000	-543.846252
94.000000	-525.932556
95.000000	-514.057007
96.000000	-509.684082
97.000000	-556.647217
98.000000	-499.888184
99.000000	-488.000854
100.000000	-513.796997

S5: Solvent accessible surface area (XVG text file).

```
# This file was Created by:
#                   :-) GROMACS - gmx sasa, VERSION 5.4.1 (-:
#
# Executable:   /usr/local/gromacs/bin/gmx
# Data prefix:  /usr/local/gromacs
# Command line:
#   gmx sasa -f md.xtc -s md.tpr -n index.ndx -o area.xvg -odg dgsolv.xvg
# gmx sasa is part of G R O M A C S:
#
# GROup of MAchos and Cynical Suckers
#
@    title "Solvent Accessible Surface"
@    xaxis  label "Time (ps)"
@    yaxis  label "Area (nm\S2\N)"
@TYPE xy
@ view 0.15, 0.15, 0.75, 0.85
@ legend on
@ legend box on
@ legend loctype view
@ legend 0.78, 0.8
@ legend length 2
@ s0 legend "Total"
      0.000  256.441
      1.000  275.591
      2.000  277.531
      3.000  276.804
      4.000  278.069
      5.000  283.827
      6.000  282.750
      7.000  281.204
      8.000  283.733
      9.000  284.015
     10.000  284.314
     11.000  284.185
     12.000  284.008
     13.000  281.958
     14.000  278.948
     15.000  280.026
     16.000  280.271
     17.000  282.285
     18.000  279.039
     19.000  280.588
     20.000  280.922
     21.000  283.350
     22.000  283.784
     23.000  281.665
     24.000  281.582
     25.000  283.544
     26.000  283.862
     27.000  277.287
     28.000  281.228
     29.000  280.881
     30.000  280.331
     31.000  280.468
     32.000  276.811
     33.000  278.778
     34.000  277.904
     35.000  278.138
     36.000  275.727
     37.000  281.710
     38.000  281.112
     39.000  278.776
     40.000  279.055
     41.000  279.123
     42.000  283.621
     43.000  281.119
     44.000  280.828
     45.000  279.372
     46.000  281.167
     47.000  285.134
     48.000  282.261
     49.000  282.544
     50.000  284.487
```

51.000	284.169
52.000	285.884
53.000	284.856
54.000	276.095
55.000	282.616
56.000	282.632
57.000	284.738
58.000	282.054
59.000	281.136
60.000	282.713
61.000	280.192
62.000	283.394
63.000	281.230
64.000	282.305
65.000	281.807
66.000	282.776
67.000	282.444
68.000	281.656
69.000	283.988
70.000	282.464
71.000	283.508
72.000	281.075
73.000	285.271
74.000	276.408
75.000	282.272
76.000	281.730
77.000	278.486
78.000	276.500
79.000	277.965
80.000	276.910
81.000	283.030
82.000	280.669
83.000	283.997
84.000	280.081
85.000	279.289
86.000	280.036
87.000	279.125
88.000	281.968
89.000	282.902
90.000	278.370
91.000	279.578
92.000	277.407
93.000	284.369
94.000	275.369
95.000	282.146
96.000	281.487
97.000	277.821
98.000	281.030
99.000	282.031
100.000	283.806

S6: H-bonds (XVG text file).

```
# This file was Created by:
#                   :-) GROMACS - gmx hbond, VERSION 5.4.1 (-:
#
# Executable:   /usr/local/gromacs/bin/gmx
# Data prefix:  /usr/local/gromacs
# Command line:
#   gmx hbond -f md.xtc -s md.tpr -n index.ndx -num hbond.xvg
# gmx hbond is part of G R O M A C S:
#
# Great Red Owns Many ACres of Sand
#
@    title "Hydrogen Bonds"
@    xaxis  label "Time (ps)"
@    yaxis  label "Number"
@TYPE xy
@ view 0.15, 0.15, 0.75, 0.85
@ legend on
@ legend box on
@ legend loctype view
@ legend 0.78, 0.8
@ legend length 2
@ s0 legend "Hydrogen bonds"
@ s1 legend "Pairs within 0.35 nm"
         0          25          21
         1          25          16
         2          24          20
         3          22          16
         4          24          17
         5          27          13
         6          24          12
         7          23          18
         8          26          11
         9          25          20
        10          26          11
        11          24          14
        12          25          13
        13          26          14
        14          25          15
        15          26          11
        16          27          11
        17          28           9
        18          25          16
        19          26          16
        20          22          20
        21          25          10
        22          20          19
        23          23          17
        24          22          16
        25          24          13
        26          24          21
        27          26          21
        28          27          11
        29          28          16
        30          28          18
        31          25          18
        32          25          17
        33          28          19
        34          23          20
        35          23          17
        36          25          13
        37          26          17
        38          27          20
        39          25          17
        40          23          16
        41          28          15
        42          24          12
        43          23          18
        44          20          23
        45          26          19
        46          25          20
        47          24          21
        48          24          19
        49          26          15
```

50	26	13
51	24	18
52	24	15
53	27	19
54	27	13
55	28	17
56	26	11
57	28	9
58	26	13
59	25	13
60	24	16
61	23	18
62	25	19
63	26	23
64	29	19
65	22	17
66	25	14
67	21	19
68	22	17
69	24	12
70	28	16
71	26	22
72	23	18
73	24	20
74	25	19
75	24	23
76	22	16
77	26	16
78	22	16
79	23	17
80	22	16
81	26	13
82	23	11
83	22	19
84	21	20
85	21	17
86	21	14
87	24	18
88	24	17
89	24	15
90	20	14
91	24	21
92	20	22
93	18	24
94	20	15
95	20	17
96	19	16
97	20	22
98	22	20
99	24	18
100	24	14

S7-Density (XVG text file).

```
# This file was Created by:
#                   :-) GROMACS - gmx energy, VERSION 5.4.1 (-:
#
# Executable:   /usr/local/gromacs/bin/gmx
# Data prefix:  /usr/local/gromacs
# Command line:
#   gmx energy -f md.edr -o density.xvg
# gmx energy is part of G R O M A C S:
#
# God Rules Over Mankind, Animals, Cosmos and Such
#
@    title "GROMACS Energies"
@    xaxis  label "Time (ps)"
@    yaxis  label "(kg/m^3)"
@TYPE xy
@ view 0.15, 0.15, 0.75, 0.85
@ legend on
@ legend box on
@ legend loctype view
@ legend 0.78, 0.8
@ legend length 2
@ s0 legend "Density"
    0.000000  1020.265076
    1.000000  1019.249146
    2.000000  1020.320068
    3.000000  1019.922363
    4.000000  1021.764404
    5.000000  1022.490479
    6.000000  1018.625977
    7.000000  1020.733215
    8.000000  1020.617554
    9.000000  1017.794128
   10.000000  1020.295349
   11.000000  1016.631592
   12.000000  1021.195374
   13.000000  1018.442688
   14.000000  1019.290466
   15.000000  1022.752319
   16.000000  1020.745789
   17.000000  1020.824890
   18.000000  1023.397888
   19.000000  1020.855774
   20.000000  1018.906250
   21.000000  1021.466492
   22.000000  1022.543518
   23.000000  1024.231201
   24.000000  1019.811951
   25.000000  1016.916809
   26.000000  1019.257935
   27.000000  1019.002014
   28.000000  1018.385803
   29.000000  1019.598755
   30.000000  1020.334290
   31.000000  1017.772034
   32.000000  1019.757324
   33.000000  1019.838074
   34.000000  1018.770142
   35.000000  1018.035095
   36.000000  1022.099792
   37.000000  1021.321838
   38.000000  1020.584595
   39.000000  1017.924500
   40.000000  1020.349548
   41.000000  1021.388611
   42.000000  1020.231873
   43.000000  1020.554443
   44.000000  1021.873413
   45.000000  1020.917419
   46.000000  1020.039917
   47.000000  1020.789490
   48.000000  1017.996216
   49.000000  1023.078003
   50.000000  1022.956116
```

51.000000	1019.453003
52.000000	1023.267273
53.000000	1020.846924
54.000000	1020.218689
55.000000	1016.716858
56.000000	1020.208862
57.000000	1022.105530
58.000000	1019.736328
59.000000	1020.999268
60.000000	1020.172913
61.000000	1023.542053
62.000000	1022.007996
63.000000	1019.963989
64.000000	1018.075012
65.000000	1016.002808
66.000000	1021.147949
67.000000	1022.944214
68.000000	1019.669250
69.000000	1020.230896
70.000000	1023.924194
71.000000	1022.229919
72.000000	1019.613647
73.000000	1023.123535
74.000000	1021.941101
75.000000	1019.969055
76.000000	1021.814392
77.000000	1022.745911
78.000000	1019.932495
79.000000	1022.913635
80.000000	1018.147339
81.000000	1019.716370
82.000000	1021.378235
83.000000	1020.393005
84.000000	1019.717590
85.000000	1020.701660
86.000000	1019.162476
87.000000	1020.543579
88.000000	1017.679871
89.000000	1019.642029
90.000000	1021.202637
91.000000	1019.675659
92.000000	1019.979919
93.000000	1020.365173
94.000000	1023.906189
95.000000	1020.777100
96.000000	1021.701172
97.000000	1019.632019
98.000000	1020.430664
99.000000	1023.175598
100.000000	1019.247131
